# Integrative analysis of the gut microbiota and metabolome in rats treated with rice straw biochar by 16S rRNA gene sequencing and LC/MS-based metabolomics

**DOI:** 10.1038/s41598-019-54467-6

**Published:** 2019-11-28

**Authors:** Jie Han, Jun Meng, Shuya Chen, Chuang Li

**Affiliations:** 1 Key Laboratory of Zoonosis of Liaoning Province, College of Animal Science and Veterinary Medicine, Shenyang Agricultural University, Dongling Road 120, Shenyang, Liaoning Province 110866 P.R. China; 2 Liaoning Biochar Engineering & Technology Research Center, Agronomy College, Shenyang Agricultural University, Dongling Road 120, Shenyang, Liaoning Province 110866 P.R. China

**Keywords:** Microbial communities, Nutrition

## Abstract

The intestinal microbiota contributes to host metabolism and health. This study aimed to assess the effects of biochar on cecal microbiome-related metabolic changes in rats. Rats were orally administered rice straw biochar (RSB) at 1120 mg/kg body weight for 5 weeks. Cecal samples were analyzed to perform metabolic and microbial profiling via a combination of 16S rRNA gene sequencing and LC/MS techniques. We observed a significant influence of RSB in shaping the cecal bacterial community, including some potentially beneficial members of phylum *Firmicutes* belonging to *unclassified Lachnospiraceae*, *Oscillibacter*, and *Clostridium XlVa* and *IV*, as well as the depletion of some opportunistic pathogens belonging to *Prevotella, Bacteroides* and *Paraprevotella*. The metabolomic analysis revealed distinct changes in the cecal metabolic phenotype, including lower levels of L-isoleucine, indole-3-acetic acid, benzoic acid, and tetradecanoic acid as well as higher levels of L-phenylalanine, L-glutamate, 3-phenylpropanoic acid, chenodeoxycholic acid, cholic acid, 7-dehydrocholesterol, (5Z, 8Z, 11Z, 14Z, 17Z)-eicosapentaenoic acid, 11-deoxycorticosterone and retinol, which are mainly involved in the metabolic pathways of linoleic acid, amino acid and steroid hormone biosynthesis. Correlation analysis revealed a positive association of *unclassified Lachnospiraceae, Oscillibacter* and *Clostridium IV* with 3-phenylpropanoic acid, L-phenylalanine, L-glutamate, 11-deoxycorticosterone and 7-dehydrocholesterol. These results confirm that the gut microbiome is altered and may be critical for good performance under RSB application by interacting with metabolism.

## Introduction

The intestinal microbiota is composed of an extremely large number of different bacteria that produce a variety of metabolites, are exclusively responsible for microbe selection and are involved in important metabolic functions, such as the biosynthesis and biotransformation of amino acids and bile acid^1^. Factors such as changes in nutritional interventions, host condition, radiation and toxicological insult can induce microbial regulation^2^ and affect host health. Therefore, the integrative analysis of gut microbiota and metabolite profiles can help to understand the changes in the host’s physiological condition under the aforementioned types of interference.

Carbonaceous adsorbents have been employed worldwide to improve animal performance as dietary interventions^3,4^ and lead to profound microbial modulation^5,6^. These adsorbents, with inhomogeneous microporous structures, can supply appropriate habitats for microorganisms^7^. Biochar is one kind of carbonaceous adsorbent, produced by the degradation of organic matter from agricultural waste in an oxygen-limited, anaerobic environment^8^. This productive process makes biochar rich in micronutrients and forms a large surface area and more optimal macropores. All of these characteristics contribute to the supply of more spacious sites and nutrition for microorganisms, contributing to their proliferation or providing “refuge”^9,10^. In China, biochar possesses the outstanding advantages of territorially unlimited sources of plant-derived raw material, a reasonable price and environmentally friendly production process. The regulation of microorganism activity and abundance by biochar has been well documented^11–13^. China is a large agricultural country where there is considerable livestock breeding, in which the utilization of biochar as a dietary supplement could be highly significant. The use of biochar could not only help to realize new feed resource development but could also improve the rational utilization of agricultural waste. However, an understanding of the effect of biochar on the metabolite profile related to the gut microflora is lacking. Moreover, considering the numerous unculturable gut bacteria, the cultural approach adopted in previous studies has suffered from the underestimation of the actual microbial community^14^. Hence, there is the need to obtain an overall view of the changes in metabolite profiles related to the gut microbiota under the inclusion of biochar in the diet, which will contribute to better understanding the beneficial effect of biochar on animal well-being^15^.

High-throughput sequencing is an accurate and powerful approach for obtaining detailed information on the microbiome^16^. It can transcend the limitations of traditional culture strategies for some of the unculturable gut microflora *in vitro*. Metabolomics represents an unprecedented approach for detecting the often-subtle differences in a broad range of metabolites in tissues or biofluids. The metabolomics technique enables the identification of the global metabolic response between the host and its gut commensal microbiota^17^. The application of metabolomics for assessing the effect of nutrition interventions is effective, especially when metabolic changes easily escape detection by traditional hypothesis-led approaches^18^. Regarding the relationship between the gut microbiota and the host metabolic phenotype, a nontargeted metabolomic study provides an effective approach for resolving the metabolic phenotypic variations related to gut microbiota perturbations, about which many research questions remain^19^.

Recently, the changes in growth performance, intestinal mucosal conditions, and the cecal microbial community induced by RSB application in rats were examined^20^. In this study, we probe the cecal microbiome-related metabolic changes in rats following the dietary inclusion of rice straw biochar (RSB) by employing high-throughput sequencing and liquid chromatography coupled to mass spectrometry (LC-MS).

## Results

### RSB-induced changes in the cecal bacterial microbiome

**Figure 1:**
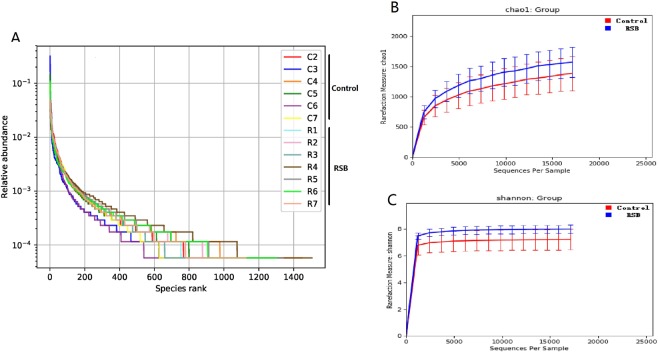
Changes in the richness and diversity of the cecal microbiota as a result of RSB treatment. (**A**) The OTU rank abundance of the cecal microbiota in rats. (**B**) Bacterial richness estimated from the Chao 1 value; (**C**) bacterial diversity estimated from the Shannon index. C2~C7 indicate rats from the control group; R1~R7 indicate rats from the RSB group.

The high-quality sequences were clustered into OTUs according to a cut-off of 97% sequence similarity. The OTU rank abundance in the RSB group exhibited a gentler slope and wider distribution on the horizontal axis, indicating that both more diversity and a more uniform distribution were induced in the cecal microbiota in response to RSB addition (Fig. 1A). The Chao1 estimation reflects the ecological species richness of the bacterial community and primarily highlights species numbers, and the Shannon index represents and correlates positively with the species distribution. The Chao1 index (average values of 1561 vs 1364, P < 0.05) (Fig. 1B) and the Shannon index (average values of 8.01 vs 7.31, P < 0.05) (Fig. 1C) in the RSB group were higher than those in the control group, indicating that RSB addition supported significantly more richness and microbial diversity in the cecum of the rats.

**Figure 2:**
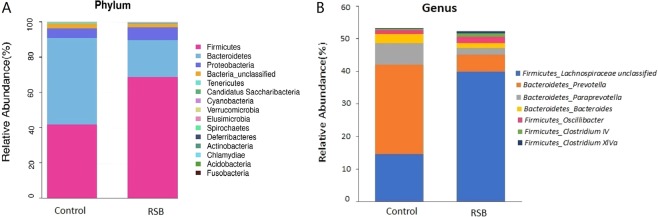
Effect of RSB addition on the changes in microbial taxa. (**A**) Relative gut microbiota abundance at the phylum level in the control and RSB groups. (**B**) Relative abundance rates of significant changes in taxa in the control and RSB rats.

The taxon summary revealed distinct changes in the gut microbial composition in response to RSB administration. In terms of the assignment at the phylum level, 15 phyla were detected in all samples, and the dominant phyla were *Firmicutes*, *Bacteroidetes*, *Proteobacteria*, and *unclassified Bacteria*, with average relative abundances in the RSB group of 68.61%, 21.01%, 7.46% and 1.79%, respectively (Fig. 2A). More importantly, the abundance of phylum *Firmicutes* in the RSB group was markedly increased by 64.18% (*P* < 0.05), whereas the proportion of *Bacteroidetes* decreased by 57.03% (*P* < 0.05) compared to those in the control group. At the genus level, the taxa whose relative abundance values were significantly affected by RSB included unclassified *Lachnospiraceae*, unclassified *Ruminococcaceae*, *Prevotella*, and unclassified *Porphyromonadaceae*, with proportions in the RSB group of 39.92%, 10.90%, 5.14%, and 5.11%, respectively, among the 122 genera identified (Fig. 2B). The percentages of unclassified *Lachnospiraceae* (34.78% of all reads, 1079 OTUs), *Oscillibacter* (1.19% of all reads, 37 OTUs), *Clostridium IV* (0.84 of all reads, 26 OTUs) and *Clostridium XlVa* (0.42% of all reads, 13 OTUs) in the RSB group were markedly increased by 172.68% (*P* < 0.05), 67.48% (*P* < 0.05), 117.5% (*P* < 0.05) and 436.36% (*P* < 0.05), respectively. Conversely, the proportions of *Prevotella* (7.58% of all reads, 235 OTUs), *Paraprevotella* (0.77% of all reads, 24 OTUs) and *Bacteroides* (0.55% of all reads, 17 OTUs) were decreased by 81.23% (*P* < 0.05), 46.60% (*P* < 0.05), and 69.54% (*P* < 0.05), respectively, compared to the control group.

### RSB-induced gut metabolic profiling analysis

**Figure 3:**
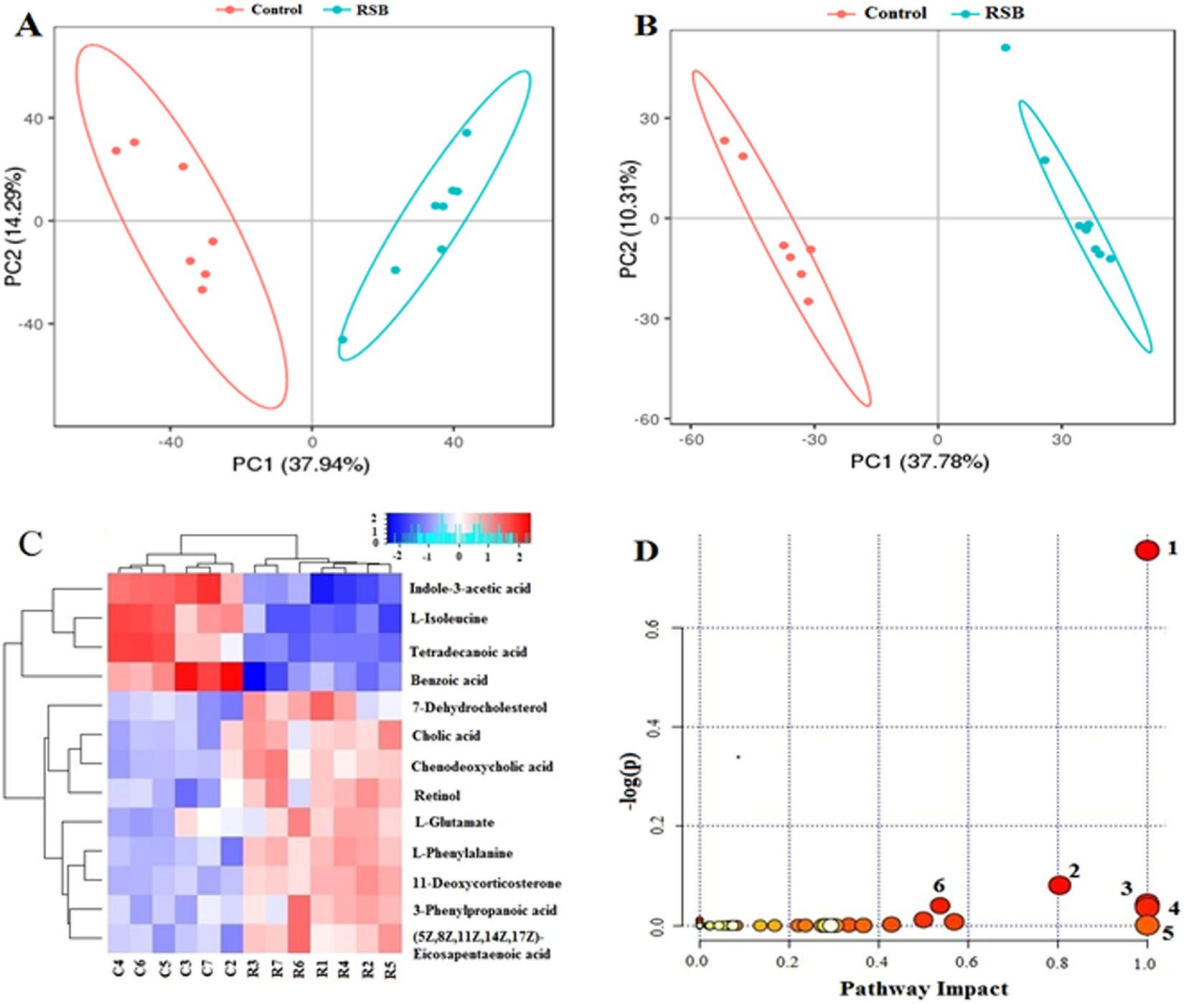
Multivariate statistical analysis, heat map and summary of metabolome pathways. (**A**) PCoA score plot of metabolite profiling between the control and RSB groups. (**B**) PLS-DA score plots of cecal samples from the control and RSB groups. (**C**) Heat map summarizing the fold changes in significantly altered metabolites in the cecal samples. Red and blue represent higher and lower concentrations of metabolites, respectively. (**D**) The impact of pathways between the control and RSB groups. Mapping of the relative concentrations of metabolites to the metabolome indicating the contributions of the impact of metabolic pathways. Node color indicates significance based on p-values, and node size indicates the significance of pathway impact. Significantly impacted pathways include (1) linoleic acid metabolism, (2) steroid hormone biosynthesis, (3) D-glutamine and D-glutamate metabolism, (4) valine, leucine and isoleucine biosynthesis, (5) alpha-linolenic acid metabolism, and (6) phenylalanine metabolism.

Multivariate analysis methods including PCA and PLS-DA were subsequently employed to reveal the clustering trends of each group. Each point in the PCA score plot represents an individual sample, and the samples are divided into blocks suggesting that the two clusters are clearly divided, representing the two groups with different metabolic profiles (Fig. 3A), thus allowing the visualization of the general changes in the cecal metabolic profile after RSB application. The supervised pattern recognition of PLS-DA focuses on the actual class-discriminating variations and shows better separation compared to the unsupervised PCA. Every dot in this score plot represents an observed sample, and the distance between two dots reflects the similarity of the metabolite composition. The score plot of PLS-DA in Fig. 3B reveals good fitness and high predictability of this model with high statistical values of Q^2^ = 0.916 and R^2^ = 0.998, indicating significant changes in the cecal metabolomes in response to RSB treatment with good clustering of the RSB group on the right and the control group on the left.

The potential identified compounds were generated using the program metaX according to the following procedure: comparison of accurate MS and MS^E^ fragments with the metabolites searched in the freely accessible HMDB database by detecting their molecular weights and elemental compositions. Compounds without information for the given mass fragment were removed from the candidate list according to the possible fragment mechanisms, and only the most likely compounds were reserved. To select the potential biomarkers responsible for this difference, the variable importance for projection (VIP) parameter combined with the p-value and fold change was used to select the variables with the most significant contribution in discriminating cecal compounds between the two groups. Fifteen metabolites with a critical VIP > 1.0, p value < 0.05 and fold change ≥1.2 or ≤0.833 were identified in the cecal samples of rats treated with RSB, and the results are listed in Table 1. The variation tendencies of these metabolites are shown in a heat map in Fig. 3C. Four metabolites were significantly decreased, including tetradecanoic acid, L-isoleucine, benzoic acid and indole-3-acetic acid. Nine metabolites were significantly enriched in the RSB addition group, including L-phenylalanine, L-glutamate, 3-phenylpropanoic acid, cholic acid, (5Z, 8Z, 11Z, 14Z, 17Z)-eicosapentaenoic acid, chenodeoxycholic acid, 11-deoxycorticosterone, 7-dehydrocholesterol and retinol. These metabolites influenced by RSB addition were mainly involved in pathways such as steroid hormone biosynthesis and amino acid transport and protein synthesis (phenylalanine metabolism, arginine biosynthesis and valine, leucine and isoleucine biosynthesis) (Fig. 3D).

**Table 1:** Identified potential biomarkers of cecal samples between the control and RSB treatment groups.

Metabolites	RT(min)	*m/z*	VIP	Formula	Trend	Related pathway
L-Phenylalanine	3.56	148	1.90	C_9_H_11_NO_2_	↑**	Phenylalanine metabolism
L-Glutamate	4.30	130	1.24	C_5_H_9_NO_4_	↑**	Arginine biosynthesis
L-Isoleucine	0.80	132	2.37	C_6_H_13_NO_2_	↓*	Valine, leucine and isoleucine metabolism
3-Phenylpropanoic acid	6.87	151	1.88	C_9_H_10_O_2_	↑**	Phenylalanine metabolism
Benzoic acid	6.44	123	1.57	C_7_H_6_O_2_	↓**	Phenylalanine metabolism
Chenodeoxycholic acid	8.27	415	1.64	C_24_H_40_O_4_	↑**	Primary bile acid biosynthesis
Indole-3-acetic acid	4.75	176	1.02	C_10_H_9_NO_2_	↓**	Tryptophan metabolism
Tetradecanoic acid	4.25	267	2.30	C_14_H_28_O_2_	↓**	Fatty acid biosynthesis
Cholic acid	8.15	391	1.53	C_24_H_40_O_5_	↑**	Primary bile acid biosynthesis
7-Dehydrocholesterol	9.70	385	1.97	C_27_H_44_O	↑**	Steroid biosynthesis
(5Z, 8Z, 11Z, 14Z, 17Z)-Eicosapentaenoic acid	7.45	307	1.92	C_20_H_34_O_2_	↑**	Biosynthesis of unsaturated fatty acids
11-Deoxycorticosterone	6.08	331	2.80	C_21_H_30_O_3_	↑**	Steroid hormone biosynthesis
Retinol	7.95	287	1.56	C_20_H_30_O	↑**	Retinol metabolism

↑: upregulated, ↓: downregulated; *P < 0.05, **P < 0.01.

### Correlation between the gut microbiome and metabolome

**Figure 4:**
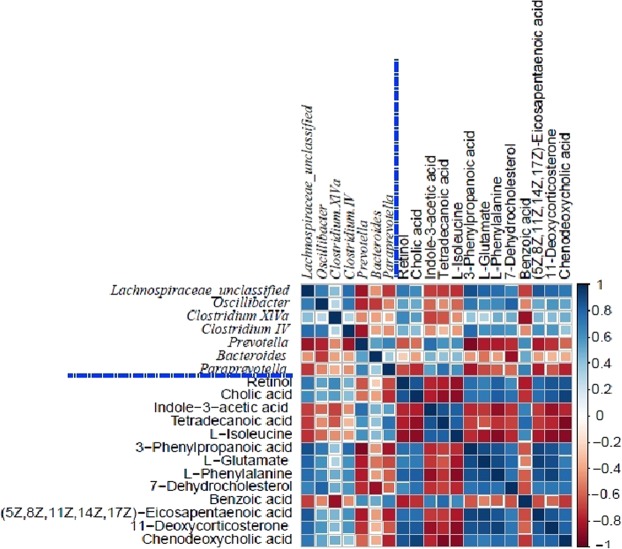
Correlation analysis of certain cecal microbiota genera and altered metabolites. Heat map summarizing the correlations of gut microbial genera and metabolites showing differences between the control and RSB groups.

Figure 4 shows the functional relationships of the altered gut microbiota and disturbed cecal metabolites following RSB application based on the Spearman correlation coefficients (|r| > 0.5, p < 0.05). Notably, unclassified *Lachnospiraceae, Oscillibacter, Clostridium IV* and *Clostridium XlVa* of phylum *Firmicutes* identified upon RSB application showed similar trends of correlation. The relative abundance of the predominant RSB-enriched gut bacterial genus of unclassified *Lachnospiraceae* was positively associated with the concentrations of nine increased metabolites, including cholic acid, 7-dehydrocholesterol, 11-deoxycorticosterone, retinol, chenodeoxycholic acid, L-phenylalanine, L-glutamate, 3-phenylpropanoic acid and (5Z, 8Z, 11Z, 14Z, 17Z)-eicosapentaenoic acid, and negatively correlated with the concentrations of four metabolites showing decreasing trends, including L-isoleucine, tetradecanoic acid, indole-3-acetic acid, and benzoic acid. when the increase in abundance was smaller, the only metabolites presenting negative associations were L-isoleucine and indole-3-acetic acid with the genus *Oscillibacter*. *Clostridium IV* and *Clostridium XlVa* possessed the same positive correlations with cholic acid and retinol. Interestingly, the relative abundances of several RSB-depleted opportunistic pathogens in phylum *Bacteroidetes* belonging to the genera *Prevotella, Bacteroides* and *Paraprevotella* showed opposite trends to those of the genera of phylum *Firmicutes. Prevotella* and *Paraprevotella* presented negative correlations with the same nine metabolites 93-phenylpropanoic acid, cholic acid, retinol, L-glutamate, L-phenylalanine, 7-dehydrocholesterol, chenodeoxycholic acid, (5Z, 8Z, 11Z, 14Z, 17Z)-eicosapentaenoic acid and 11-deoxycorticosterone) and positive correlations with four metabolites (tetradecanoic acid, L-isoleucine, benzoic acid, and indole-3-acetic acid). Our results also revealed clear correlations among some metabolites. For example, retinol was strongly positively correlated with chenodeoxycholic acid and cholic acid; chenodeoxycholic acid was correlated with cholic acid; and 11-deoxycorticosterone was associated with L-phenylalanine.

## Discussion

Here, we investigated the effect of RSB addition on cecal microbiota-related metabolic phenotype alterations in rats. Our findings showed that RSB treatment improved cecal microbial richness, which was proven by the higher OTU rank abundance and the values of the Chao 1 and Shannon indexes. Similarly, the microbial diversity of the cecal contents was improved according to the notable abundance changes in phylum *Firmicutes* in the genera of unclassified *Lachnospiraceae, Oscillibacter*, *Clostridium IV*, and *Clostridium XlVa* as well as phylum *Bacteroidetes* in the genera *Prevotella, Paraprevotella*, and *Bacteroides*. These components were verified in our previous report^20^. These results indicated that RSB inclusion induced more specific flora OTUs and a positive impact on the diversity of the ecosystem of the gut bacterial community. Additionally, PLS-DA revealed 13 cecal compounds that were the most important in differentiating the RSB and control groups, including L-phenylalanine, L-glutamate, L-isoleucine, 3-phenylpropanoic acid, benzoic acid, tetradecanoic acid, chenodeoxycholic acid, cholic acid, 7-dehydrocholesterol, 11-deoxycorticosterone, retinol, (5Z, 8Z, 11Z, 14Z, 17Z)-eicosapentaenoic acid and indole-3-acetic acid.

Increasing evidence suggests that metabolite profiles related to the gut microbiome that change under nutritional interventions are important factors in the modulation of tissue functions and achieving health improvement^1^. Accordingly, positive associations between the compounds 3-phenylpropanoic acid and benzoic acid and the microbiota components of *Clostridium, Oscillibacter*, and *unclassified Lachnospiraceae* were observed in our work. 3-Phenylpropionic acid and benzoic acid are major polyphenol compounds identified in the digestive tract^21^. It is not only the interactions between the gut microbiota and polyphenols but also the mechanisms of their interactions and the effects on host health that have been investigated^22^. Phylum *Firmicutes* produces a smaller number of glycan-degrading enzymes involved in polyphenol degradation than *Bacteroidetes*^23^; thus, the gut microbiota might be reshaped due to the intake of different polyphenols. Conversely, phenolic compounds have the ability to alter the gut microbiota balance of *Bacteroides/Firmicutes*^24^, which was supported by our observation of an increased ratio of *Firmicutes* to *Bacteroidetes* among the cecal bacteria phyla under RSB inclusion, and this change in the bacterial ratio has been considered to contribute to the improvement of health status in many studies^25,26^. Moreover, genus *Clostridium* is mainly involved in the metabolism of many phenolics^27^. Its positive associations with 3-phenylpropanoic acid and benzoic acid demonstrated that this microbial population may be closely associated with the generation and bioconversion of these polyphenols under RSB application. This suggested that RSB exerted beneficial effects on animal well-being, which is consistent with our previous report demonstrating the beneficial effects of the application of RSB on growth and intestinal health^20^.

Amino acids are the major building blocks of proteins and are important in the regulation of energy and protein metabolism in organisms^28^. Some amino acids, including the essential amino acids L-isoleucine and L-phenylalanine as well as the nonessential amino acid L-glutamate, were affected after RSB application in our work. The intimate relationships between amino acids and amino acid-fermenting bacteria belonging to the *Clostridium* clusters have been identified this group as the predominant amino acid-fermenting microbiota along the digestive tract of humans and animals^28^. In line with the above research, the current study showed a clear positive association of L-glutamate and L-phenylalanine with the abundance of genus *Clostridium IV* under RSB inclusion, which suggested that oral RSB supplementation might particularly impact amino acid metabolism and concomitant host nutrition and health. Previous research has uniformly supported the conclusion that animal dietary supplementation of biochar can improve intestinal villus histology^20,29^ and enhance nutrient digestibility^15^ and feed conversion^30^, which may be partly related to improvement of AA metabolism by biochar.

RSB application also changed the concentrations of some compounds participating in fat metabolism, such as tetradecanoic acid, chenodeoxycholic acid, cholic acid, 7-dehydrocholesterol, 11-deoxycorticosterone and (5Z, 8Z, 11Z, 14Z, 17Z)-eicosapentaenoic acid. Tetradecanoic acid belongs to the saturated fatty acids and is derived from the microbial hydrogenation of nonconjugated dienoic acids^31^. It has been identified in the animal digestive tract^32^ and in general triggered inflammatory bowel disease^33^. However, (5Z, 8Z, 11Z, 14Z, 17Z)-eicosapentaenoic acid is a long-chain poly-unsaturated fatty acid that contributes to regulating the triad of adiposity, inflammation, and fatty acid metabolism^34^. The positive associations of tetradecanoic acid with *Prevotella* and *Paraprevotella* demonstrate that microbial hydrogenation to synthesize tetradecanoic acid by hydrogen-producing *Prevotella* and *Paraprevotella* is suppressed and consequently decreases the risk of a gut inflammatory reaction in response to RSB treatment.

In addition, *Clostridium IV* and *XIVa*, which are members of phylum *Firmicutes*, have been demonstrated to be significantly less abundant in inflammatory bowel disease patients than in healthy individuals. These microbiota have proven to be important in maintaining the diversity of the clostridia and preventing intestinal inflammation^35^. The negative correlation of *Clostridium IV* and *XIVa* with tetradecanoic acid potentially suggests that these microbiota may contribute to the resistance of gut inflammation through the modulation of tetradecanoic acid. Cholesterol can be metabolized for the synthesis of cholic acid, chenodeoxycholic acid, 11-deoxycorticosterone and 7-dehydrocholesterol through various metabolic processes^34^. Cholic acid and chenodeoxycholic acid are the primary bile acids in most species. They play an important physiological role in boosting the absorption of vitamins and fats and regulating bile acid biosynthesis and cholesterol homeostasis^36^. 11-Deoxycorticosterone is a steroid hormone that is mainly involved in the maintenance of the blood salt balance^37^. The elevated proportions of chenodeoxycholic acid, cholic acid, 7-dehydrocholesterol, and 11-deoxycorticosterone identified in our study suggested that RSB particularly participated in the regulation of fat metabolism and sterol biosynthesis. Retinyl phosphate plays an important role in the synthesis of cell membrane proteins and participates in the maintenance of cell membrane integrity. Retinyl phosphate also participates in the synthesis of steroid hormones and affects the growth and development of the host^38^. Our observation of increased concentrations of retinol combined with elevated proportions of 11-deoxycorticosterone and 7-dehydrocholesterol in the steroid biosynthesis pathways after RSB application further indicated that RSB may mainly affect the synthesis of steroid hormones to modulate host growth and development.

In summary, our results from 16S rRNA gene sequencing showed that RSB application significantly changed the gut microbiota community in terms of its diversity and composition. Additionally, RSB altered the metabolic phenotype according to LC-MS metabolomic analysis, which revealed major roles in linoleic acid metabolism, steroid hormone biosynthesis, amino acid transport and protein synthesis. In addition, the correlation analysis revealed relationships between some differentially abundant gut microbes and the candidate metabolites. In general, our results confirmed that the gut microbiome is altered and contributes to performance by interacting with metabolism under RSB application.

## Materials and Methods

### Rice straw biochar preparation and characterization

The details of the preparation and the characteristics of RSB have been described previously^20^.

### Animals and experimental design

The animals and experimental design used in our study were consistent with our previous report^20^. Specific pathogen-free grade female Wistar rats at 5 weeks of age weighing 190–210 g that were commercially obtained from the Liaoning Experimental Animal Resources Center (Benxi, China), were used in the current study. All the animals were housed under standard laboratory conditions (22 ± 2 °C, 53% ± 2% relative humidity, 12:12 light/dark cycle) with *ad libitum* access to chow and water. All the rats were randomly divided into control and RSB groups of 10 rats per group after acclimation for one week. An RSB solution with a concentration of 112 mg/mL was prepared using ultrapure deionized water and administered to the rats via oral gavage at a dose of 1120 mg/kg body weight (BW) daily for 5 weeks. The control group received an equivalent amount of ultrapure deionized water instead. The gavage volume of 1 mL/100 g BW was adjusted according to BW once per week. The oral dose of RSB was selected in reference to the results obtained from our previous study^15^ by using a dose conversion formula for rats^39^. Following anesthesia and cervical dislocation, samples of the cecal contents of the rats were immediately collected and snap-frozen in liquid nitrogen to halt metabolism, then stored at −80 °C prior to the metabolic profiling and microbial community analyses. The Animal Ethics Committee of Shenyang Agricultural University approved this study, and all management and experimental procedures were conducted according to the Guidelines for the Care and Use of Animals of Shenyang Agricultural University.

### Bacterial sequencing and analysis

The bacterial sequencing analysis performed in our study was consistent with our previous report^20^. Total DNA was extracted from the cecal contents by utilizing the E.Z.N.A. Stool DNA kit (D4015, Omega, Inc., USA). The V3-V4 region of the 16S rRNA gene was selected for subsequent pyro-sequencing. PCR amplification was performed with slightly modified versions of the primers 338F (5′-ACTCCTACGGGAGGCAGCAG-3′) and 806R (5′-GGACTACHVGGGTWTCTAAT-3′)^40^. PCR amplification reactions were performed in a total volume of 25 μL, and the reaction mixtures contained 25 ng of template DNA, 12.5 μL of PCR premix, 2.5 μL of each primer, and PCR-grade water to adjust the volume. The PCR conditions for the amplification of the prokaryotic 16 S fragments consisted of initial denaturation at 98 °C for 30 s, followed by 35 cycles of denaturation at 98 °C for 10 s, primer annealing at 54 °C for 30 s, and extension at 72 °C for 45 s, with a final extension at 72 °C for 10 min. Then, the PCR products were visualized on a 2% agarose gel and cleaned by using AMPure XP beads (Beckman Coulter Genomics, Danvers, MA, USA) and quantified with a Qubit fluorometer (Invitrogen, Carlsbad, California, USA). The amplicon library was assessed for size and quantity by using an Agilent 2100 Bioanalyzer (Agilent, USA) with a Library Quantification Kit for Illumina (Kapa Biosciences, Woburn, MA, USA) and sequenced on the Illumina 300PE MiSeq platform. Chimeric sequences were filtered using Verseach software (V 2.3.4). The filtered sequences were clustered into operational taxonomic units (OTUs) based on a threshold of 97% sequence similarity. An analysis of UniFrac dissimilarities was calculated according to the levels of changes in the gut microbiota using R (version 3.2.1) with the vegan package.

### Sample preparation for metabolic profiling and LC-MS analysis

After the gut contents were thawed on ice, precooled 50% methanol was added to 25 mg of the samples, followed by vortexing for 1 min and incubation at room temperature for 10 min. The extraction mixture held overnight at −20 °C and centrifuged (25,000 × *g*) at 4 °C for 15 min to obtain the supernatant. Then, the supernatants were transferred to new 96-well plates and stored at −80 °C for subsequent LC-MS analysis.

Chromatographic separation was performed in an ultra-performance liquid chromatography system (UPLC) (SCIEX, California, USA) using an ACQUITY UPLC BEH C18 reversed-phase column (2.1 mm × 100 mm, 1.7 μm) (Waters, Massachusetts, USA) with a constant column temperature of 35 °C. After equilibration for 2 min, the column flow rate remained at 400 μL/min, and the injection volume of each sample was 4 μL. The mobile phase consisted of solvent A (25 mM ammonium acetate + 25 mM NH_3_·H_2_O) and solvent B (isopropanol:acetonitrile = 9:1 + 0.1% formic acid). The UPLC elution conditions were optimized and set as follows: 0~0.5 min, 95% B; 0.5~9.5 min, 95% to 65% B; 9.5~10.5 min, 65~40% B; 10.5~12 min, 40% B; 12~12.2 min, 40~95% B; 12.2~15 min, 95% B.

A Triple TOF5600+ high-resolution tandem mass spectrometer (SCIEX California, USA) was used to detect the eluted metabolites. Electrospray ionization in both positive and negative modes was employed to introduce the sample to the mass spectrometer. The curtain gas was set at 30 PSI with ion source gas 1 and gas 2 both at 60 PSI. The capillary voltage and cone voltages were set at 2000 V and 40 V for positive ion mode and 1000 V and 40 V for negative ion mode, respectively. The desolation gas was set at 450 L/h at 350 °C; the cone gas was set at 50 L/h; and the source temperature was set at 120 °C. The TOF mass range was from 60 to 1200 Da. The survey scans were acquired over 0.15 s, and as many as 12 production scans were collected if they exceeded a threshold of 100 counts per second (counts/s). Four time bins were summed for each scan at a impulsator frequency value of 11 kHz with four-anode/channel detection.

### Data processing and statistical analysis

LC/MS raw data were converted into mzXML format and processed using XCMS software to detect and align features. The XCMS analysis provided a matrix including peak picking, peak grouping, retention time (RT) correction, second peak grouping, and annotation of isotopes for every sample. Quality control samples (QCs) were injected at the beginning of the analysis and detected after every 5 samples to evaluate the stability of the LC/MS throughout acquisition. The features detected in less than 50% of QCs or 80% of biological samples were removed; the combination of the RT and m/z data was used to identify each ion. The intensity data of each peak were recorded and further preprocessed with the in-house software metaX. Outlier detection and batch effect evaluation were performed through principal component analysis (PCA) by using the preprocessed dataset. Quality control-based robust LOESS signal correction was fitted to the QC data with respect to the order of injection to minimize signal intensity drift over time. The relative standard deviations of the metabolic features exceeding 30% were removed, and the P value was adjusted for multiple tests using a false discovery rate (Benjamini–Hochberg). Supervised PLS-DA was conducted with metaX to discriminate the different variables between groups. The VIP value was calculated, and a cut-off value of 1.5 was used to select important features. The online HMDB database (http://www.hmdb.ca) was used to annotate the metabolites by matching the exact molecular mass data (m/z) of the samples with those from the database. The correlations between the cecal compounds and bacterial profiles were assessed with Spearman’s correlation test using R (version 3.2.1) with the vegan package.

### Statistical analysis

The results were statistically analyzed using IBM SPSS statistical software (version 22.0). The significant differences between the control and RSB supplementation groups were analyzed by one-way ANOVA, followed by a two-tailed Student’s t-test. *P* < 0.05 was considered to indicate a statistically significant difference.
